# Highly transparent elastic optical microcavities for interferometric mapping of cell mechanics

**DOI:** 10.1364/BOE.572738

**Published:** 2025-10-22

**Authors:** F. Busse, J. H. Booth, N. M. Kronenberg, Y. Sun, A. T. Meek, A. Mischok, S. R. Pulver, M. C. Gather

**Affiliations:** 1 Humboldt Centre for Nano- and Biophotonics, Department of Chemistry and Biochemistry, University of Cologne, Cologne, Germany; 2 Centre of Biophotonics, SUPA, School of Physics and Astronomy, University of St Andrews, St Andrews, United Kingdom; 3 Centre of Biophotonics, University of St Andrews, United Kingdom; 4 School of Psychology and Neuroscience, University of St Andrews, United Kingdom; 5 Cologne Excellence Cluster on Cellular Stress Responses in Aging-Associated Disease (CECAD), University of Cologne, Cologne, Germany

## Abstract

Elastic resonator interference stress microscopy (ERISM) enables label-free, non-invasive measurements of cellular forces by detecting the cell-induced deformation of an optical microcavity using optical interference. Here, we present an improved microcavity design that utilizes a high-refractive-index elastomer, eliminating the need for the top metal layer required in traditional ERISM microcavities. This simplifies the fabrication process, offers improved control over mechanical properties, and enhances the resolution of ERISM measurements. The design is compatible with various bio-coatings, maintains long-term cell viability, and significantly improves optical transmission. This enables integrated and confocal fluorescence imaging with improved contrast over traditional ERISM microcavities.

## 1. Introduction

Cellular mechanics and mechanosensing are pivotal in a wide array of biological processes [1,2], including tissue development [3,4], disease mechanisms [5,6], cell migration [7,8], and signaling pathways [9,10]. Quantifying physiological properties such as forces at the cellular scale is essential for understanding these complex phenomena [11,12]. Common techniques employed for this purpose are traction force microscopy [13–15], elastic micropillars [16,17] and FRET-based tension sensors [18,19]. Recently, elastic resonator interference stress microscopy (ERISM) has been introduced as a powerful alternative for the measurement of cellular forces; it offers high sensitivity, is inherently non-invasive and unlike other approaches for cell force mapping does not require zero-force reference measurements [20–24].

ERISM leverages thickness-dependent changes to the optical interference within an elastic optical microcavity to detect deformations induced by cells that adhere to the top surface of this cavity and reaches nanometer-scale precision. So far, ERISM microcavities have comprised two thin gold layers (thickness ≈ 15 nm) separated by an elastomeric layer (thickness ≈ 8 µm) to provide sufficient reflection and thus adequate interference contrast for measuring deformations of the microcavity surface induced by cells. While these microcavities have proven highly effective for cell force mapping, their fabrication requires vacuum equipment for the deposition of the thin gold films. Furthermore, the fabrication process is susceptible to defects owing to the delicate nature of the gold-elastomer interface. As a result, the adhesion and ‘wetting’ of the gold on the elastomer must be enhanced via a carefully optimized surface treatment of the elastomer. This in turn leads to stiffening of the elastomer surface, complicating the analysis of cell-induced substrate deformations. Further challenges include the need to readjust the fabrication process when elastomers of different physical stiffnesses are used, and the relative opacity of the gold films, which adversely affects the signal quality of complementary imaging techniques, such as fluorescence microscopy, when combined with ERISM.

To address these limitations, we have developed a microcavity design that incorporates a high-refractive index elastomer to achieve the microcavity effect by Fresnel reflection at the elastomer-cell interface, thereby eliminating the need for an additional top gold layer. This approach significantly simplifies the fabrication process whilst preserving the essential optical properties required for sufficient interference contrast within the microcavity. Different biological systems favor different physical properties of their surroundings and the physiology and behavior of mechanosensitive cells is strongly influenced by their environment, e.g. the substrate stiffnesses [7,25–27]. By adjusting the mixing ratio of the two elastomer precursors, we can precisely adjust the stiffness of the device, without having to take the wetting characteristics of a subsequent gold layer into consideration. Additionally, the enhanced transmission of the gold-free, high-index design greatly improves the quality of fluorescence microscopy images of cells cultured on the new microcavity design.

This study presents the development and characterization of the high-index microcavities. We demonstrate their capability to measure cell-induced deformations with precision comparable to traditional gold film based microcavities. Furthermore, we show the long-term viability of cells cultured on the microcavities and illustrate their versality in cellular biomechanics applications by adjusting the device stiffness, using NIH-3t3 fibroblast cells as our model for validation. Finally, we demonstrate an improved signal-to-noise ratio of fluorescence images recorded with the new microcavity design.

## 2. Materials and methods

### 2.1. ERISM theory

ERISM is described in detail by Kronenberg *et al* [20]. In brief, for ERISM measurements, monochromatic light is used to illuminate an optical microcavity in an epi-widefield configuration. In this work, the cavity is composed of a high-refractive index elastomer (thickness ≈ 8 µm) deposited on a glass substrate coated with a thin, 80 nm-thick Ta_2_O_5_ layer. The significant refractive index difference between the elastomeric layer (*n* = 1.50 at a wavelength of 650 nm, see Supplementary Note 2) and the medium or cells above (e.g. water with *n* = 1.33 and cells with *n* ≈ 1.37) enhances the internal light reflection at the cavity-water interface. The ratio of reflected to transmitted light depends on the optical thickness of the cavity *nd* (*n*, refractive index; *d,* local cavity thickness) and the wavelength *λ* for an integer mode number *m*, where the reflection is maximal when:

(1) mλ=2nd

As a result, for a given wavelength *λ*, an interference pattern can be observed with alternating bright (high reflection) and dark (low reflection) fringes that are dependent on the local cavity thickness [28]. Due to the elastic properties of the device, the local cavity thickness is altered when cells attached to the surface exert forces to the substrate resulting in changes of the interference fringe pattern.

For a quantitative analysis, the illumination is scanned across multiple wavelengths, which in effect provides a reflectance spectrum for each pixel in the field of view. The data analysis process is thoroughly described by Liehm *et al* [21]. In short, a transfer matrix method (‘TMM’) simulation [29] is used to calculate the expected reflectance spectrum for the microcavity over the entire relevant range of microcavity thicknesses in suitable thickness steps. In our study, the layer structure used for the calculations is 500 µm glass substrate, 80 nm Ta_2_O_5_ and a thickness of elastomer in a range between 3000 nm and 15000 nm was considered, with a 1 nm step size. The positions of all minima in the reflectance spectrum are then extracted using a standard peak-finding procedure to create a database of resonant wavelengths for the range of possible microcavity thicknesses. A custom Python script is used to compare the minima extracted from the reflectance spectra to this database for each pixel in the field of view. The cavity thickness is determined as the thickness where simulation and experimental data show the best agreement.

### 2.2. Device fabrication

High-index microcavities were constructed on cleaned 24 × 24 mm^2^ No #1.5 or No #5 thickness glass substrates. An 80 nm-thick Ta_2_O_5_ layer was sputtered from >99.99% oxide targets (Angstrom Engineering). The two precursors of the phenyl silicone-based elastomer (QGel 920, CHT) were mixed in different ratios of part A and B (1:1, 1:1.33, 1:1.5, 1:2) to achieve the desired elastomer stiffness (5 kPa, 12 kPa, 19 kPa, or 34 kPa in the present study) and degassed by centrifugation at 3250 rpm for 5 minutes. The mixture was spincoated onto the Ta_2_O_5_ coated substrate in a two-step cycle; first at 300 rpm for 30s (acceleration 500 rpm/s) to wet the surface of the substrate with the elastomer, followed by either 6000 rpm for mixing ratios A:B 1:1 and 1:1.33 or 6500 rpm for mixing ratios of 1:1.5 and 1:2 (acceleration 1500 rpm/s) to obtain a final elastomer layer thickness of 8-10 µm. The elastomer was crosslinked on a hotplate at 105 °C for 60 min. The fabrication process for the gold layer-based ERISM microcavities used as a reference in this study is described by Kronenberg *et al* [20].

### 2.3. ERISM measurement

Monochromatic illumination was provided by a white light halogen source combined with a scanning monochromator (CM110, Spectral Products) for spectral filtering and a 0.6-mm wide slit for spatial filtering. The probe light was directed into an inverted fluorescence microscope (Eclipse Ti, Nikon) and focused to the back aperture of a 10x (Plan Fluor, NA 0.3, Ph1, Nikon), 20x (Plan Fluor, NA 0.45, Ph2, Nikon) or 40x (Plan Fluor, NA 0.6, Ph2, Nikon) objective to obtain collimated, widefield illumination of the microcavity. Unless stated otherwise, the 20x objective was used to perform ERISM measurements. The reflection signal was collected using the same objective and transmitted through a 50:50 beamsplitter to an sCMOS camera (Zyla 5.5, Andor). Imaging and wavelength scanning were controlled via custom LabView software. The exposure time for each wavelength image was set to 100 ms, resulting in a total measurement time of 20 seconds for a full wavelength scan from 560 to 760 nm in 1 nm-increments. The wavelength range was slightly adjusted compared to the commonly used wavelength range (550 to 750 nm) to avoid cutting off light at a dichroic mirror (cut-off wavelength 550 nm), that was implemented to combine wide-light and monochromatic illumination for simultaneous transmission and ERISM imaging. The full wavelength scan was only used in a first measurement to determine the resonance order of the interference fringes. For subsequent measurements, a reduced wavelength range of 650 to 700 nm was used to track the spectral position of a single reflectance minimum, reducing the scanning time to 5 seconds per field of view. The acquisition time was adjusted to 50 ms for measurements with the traditional gold layer microcavity.

Transmission images were automatically acquired after each measurement using a second camera (iCube NS4133BU, NET) mounted to the auxiliary microscope port. Thickness maps were generated using a custom Python code as described in Section 2.1. Displacement maps were obtained by subtracting the background from the thickness images in the ImageJ software, and for Fourier filtered maps, an FFT bandpass filter was applied to the displacement maps.

Cells areas were manually determined from phase contrast images using ImageJ. To determine the positive displacement volume, in the coating experiment, a threshold of 20 nm (10 nm for the uncoated sample) was applied to the ERISM displacement maps. In the stiffness variation experiment, different thresholds of minimal deformation were applied to account for the varying deformation depths of microcavities with different stiffnesses, namely 5 kPa > 30 nm, 12 kPa > 20 nm, 19 kPa > 17 nm, 34 kPa > 12 nm. The mean displacement was determined within the manually selected displacement area using ImageJ.

### 2.4. Cell experiments

Unless otherwise specified, a silicone structure consisting of four wells (2 × 2) with dimensions of 0.75 × 0.75 cm^2^ (prepared from removable 12 well chambers, ibidi, Cat. No. 81201) was placed atop of a microcavity. The device surface was coated with a bio-coating solution containing 94.98% Dulbecco’s phosphate buffered saline (dPBS, Gibco, Cat. No. 14190), 0.02% Gelatin (Sigma-Aldrich, G1393), and 5% fibronectin bovine plasma (Merck, Cat. No. F1141) (FibGel) and incubated for 1 h at room temperature, after which the excess coating solution was removed. NIH-3t3 cells were trypsinized from a cell culture flask, centrifuged, and resuspended in cell culture medium composed of Dulbecco’s modified eagle medium (DMEM, Gibco, Cat. No. 21969), supplemented with 10% fetal bovine serum (FBS, Gibco, Cat. No. A47361), 1% penicillin/streptomycin solution (Pen Strep, Gibco, Cat. No. 15140-122), and 1% 100× Glutamax (Gibco, Cat. No. 35050). Approximately 2500 to 3000 cells were seeded in each chamber of the microcavity in a volume of approximately 250 µL of cell medium and incubated for ≈ 24 hours prior to experiments. For ERISM and live fluorescence measurements, the microcavity was placed in an on-stage microscope incubator (OKOLAB) to maintain standard cell culture conditions (37°C, 5vol% CO_2_, 95% humidity).

For the coating experiment, different bio-coatings were applied to three microcavities with a stiffness of 12 kPa. In two of the devices, three of the wells were coated with 200 µg/mL collagen A (prepared with 200 µL of acidic PBS (pH = 4, adjusted with 0.1N HCl)) and 50 µL collagen A (Sigma Aldrich, Cat. No. L7220)), fibronectin and FibGel, respectively, while the fourth well was left uncoated as a negative control. The microcavities were incubated at 37°C for 1 hour, after which the collagen solution was washed with cell medium and the excess fibronectin and FibGel coating solutions were removed. For Matrigel coating, growth factor reduced Matrigel Matrix (354230, Corning, USA) was diluted on ice to 0.22 mg/mL in KnockOut DMEM (10829018, Gibco, USA). 250 µL of the Matrigel mixture was added to each well of a frozen microcavity (−20 °C, 3 hours), incubated at room temperature for ≈ 12 hours and stored at 4 °C for one day, before washing with cell medium.

For the time-lapse experiments, a single-well silicone structure with inner dimensions 1.60 × 1.60 cm^2^ was placed on a microcavity (stiffness, 30 kPa). Approximately 10,000 cells were added to the chamber, and the device was incubated 2 hours before starting the measurement to allow the cells to settle but to ensure the attachment process is included in the measurement. To minimize evaporation of the cell medium during the 2-day measurement period, additional water reservoirs were placed in the on-stage incubator.

### 2.5. Fluorescence microscopy

For the comparison of fluorescence measurement with gold layer-based and high-index microcavities, NIH-3t3 cells were fixed directly on the two types of devices with 4% paraformaldehyde (prepared from 10% in PBS, Thermo Scientific, Cat. No. 047317.9M) in PBS at room temperature for 10 minutes. After fixation, cells were washed with 0.05% Tween 20 (Thermo Fisher Scientific, Lot. 187799) in PBS, permeabilized using 0.1% Triton X-100 (Merck, CAS No. 9036-19-5, diluted in PBS), and blocked with 1% BSA (Sigma-Aldrich, CAS No. 9048-46-8) in PBS containing 5% donkey serum (Abbkine, Cat. No. BMS0140). Cells were stained for vinculin using a mouse anti-vinculin monoclonal antibody (Merck Millipore, Part No. 90227, purified clone 7F9, 1:100 in 1% BSA, 1 hour, room temperature (RT)), followed by secondary donkey anti-mouse AlexaFluor Plus 647-conjugated antibody (Thermo Fisher Scientific, Cat. No. A32787, 1:50 in 1% BSA, 1 hour, RT, in the dark). Simultaneously, nuclear DNA was stained with DAPI (Merck Millipore, Part No. 90229, 1:1000 in 1% BSA, 1 hour, RT, in the dark), and F-actin structures were labeled with TRITC-conjugated Phalloidin (Merck Millipore, Part No. 90228, 1:500 in 1% BSA, 1 hour, RT, in the dark).

For live-cell fluorescence experiments, the culture medium (as described in Section 2.4) was replaced with phenol red-free medium prepared using phenol red-free DMEM (Gibco, Cat. No. 31053) supplemented with 10% Fetal Bovine Serum (FBS), 1% penicillin/streptomycin solution, and 1% 100× GlutaMAX (Gibco, Cat. No. 35050). The nuclei of NIH-3t3 cells were stained with Hoechst 33342 (Thermo Fischer, CAS No. H1399, 1:2000, 1 hour, 37°C) and F-actin was labeled in parallel with SPY555 (Spirochrome, 1:300, 1 hour, 37°C) or SiR F-actin labelling (Spirochrome, 1:300, 1 hour, 37°C).

Epi-fluorescence measurements were conducted on the inverted microscope employed for ERISM measurements, equipped with a CCD camera (Nikon, DS-Qi1Mc) at the auxiliary microscope port. A 20x objective (Plan Fluor, NA 0.45, Ph1, Nikon) was used for fluorescence measurements on the fixed cells and an 40x (Plan Fluor, NA 0.6, Ph2, Nikon) for those on live cells. Z-stack fluorescence images were acquired using a confocal microscope (Stellaris 8, Leica) with a 63x oil-immersion objective (Leica, NA 1.4, Cat. No. 11506350) and a white light laser.

### 2.6. Atomic force microscopy

For AFM force-indentation measurements (FlexAFM, Nanosurf), a spherical glass bead with a diameter of 20 µm was affixed to a cantilever with a nominal force constant between 0.08–0.15 N/m (qpCONT, Nanosensors). The actual force constant was determined by thermal tuning before gluing the bead to the cantilever. The indentation profile was taken from a measurement with an ATEC-CONT cantilever (Nanosensors). Measurements were performed in concentrated buffer solution (CHAPS, 3-[(3-cholamidopropyl)dimethylammonio]-1-propane sulfonate, Roth) to minimize the electrostatic interaction between the AFM probe and the sample. Cantilever deflection was calibrated using a rigid glass substrate and a known Z-travel distance. The force-distance curves obtained for the microcavities were fitted using a corrected Hertz model [30] to determine the stiffness, assuming that the microcavity behaves as a linear elastic material and neglecting friction between the AFM indenter and the sample surface. For an elastomer mixing-ratio of 1:1.5 (A:B), the stiffness was linearly approximated as 19 kPa.

### 2.7. UV/VIS measurements

For the transmission data, a gold layer-based microcavity and a high-index elastomer microcavity (stiffness ∼5 kPa) were placed in a UV/VIS/NIR double-beam spectrometer (Agilent Cary 6000i with UMA). The transmission spectrum was recorded at an angle of 0° for a wavelength range of 400 nm to 800 nm in steps of 1 nm.

### 2.8. Ellipsometry

The optical constants of the elastomers used in the gold layer-based microcavity and the high-index elastomer microcavity (stiffness ∼5 kPa) were determined by variable angle spectroscopic ellipsometry measurements (VASE, M200, J.A. Woollam) and subsequent modeling (via CompleteEase software, J.A. Woollam) with an anisotropic model.

## 3. Results

### 3.1. ERISM concept and microcavity design

**Fig. 1.**
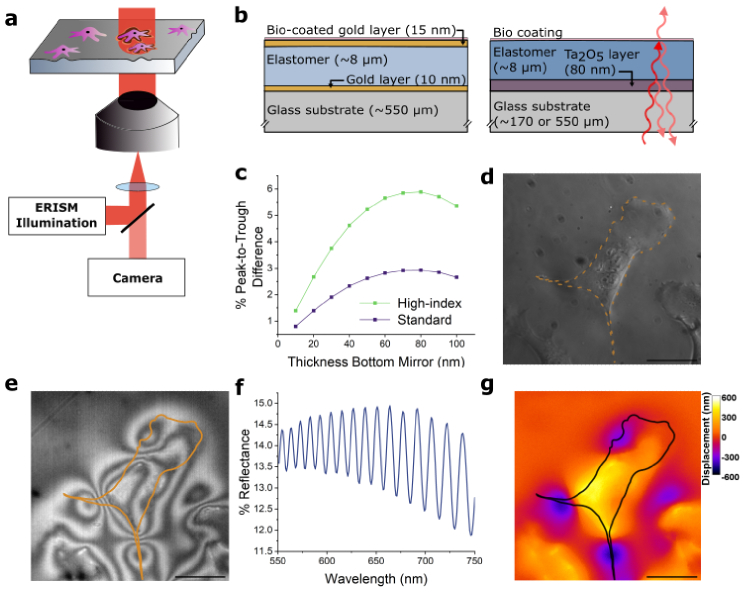
**ERISM concept and microcavity design based on a high-index elastomer. a**, Schematic representation of the ERISM setup. The microcavity is illuminated with collimated monochromatic light in an episcopic-wide-field configuration. The interference pattern formed in the ERISM microcavity is collected through the same objective, passed through a 50:50 beamsplitter, and directed to a camera. **b**, Comparison between the structure of the traditional gold mirror microcavities and the newly developed microcavities based on the high-index elastomer including a schematic light path depicting the partial reflection of light at the elastomer surfaces in the new design. **c**, Mean peak-to-trough contrast in the reflection spectra within a 550-750 nm wavelength range for a cavity without gold mirrors utilizing the previously used elastomer (Standard) and for the design with the high-index elastomer presented in this work (High-index). Data obtained by transfer matrix modelling of reflectance spectra, assuming a 500 µm glass substrate, a Ta_2_O_5_ layer with variable thickness and an 8 µm thick elastomer layer with water on top. (Simulations in vacuum provided in Supplementary Note 2.) **d**, Phase contrast image of a representative NIH-3t3 cell on top of a high-index microcavity, with cell outline shown by dashed orange line. **e**, Reflection image of the same field of view, using illumination with monochromatic light of 650 nm wavelength. Cell outline shown by orange line. **f**, Representative reflection spectrum of a high-index cavity. **g**, False color-coded displacement map calculated from the reflectance data at each pixel in (e). Color scale gives elastomer displacement in nm. Scale bars, 50 µm.

The fundamental principle underlying the optical setup for ERISM is illustrated in Fig. 1(a) (also see Section 2.1 for further details, a detailed sketch of the experimental setup including the fluorescence light path can be found in Supplementary Fig.S1). When cells apply mechanical force to the elastic optical microcavity, the elastomer surface is deformed and the local cavity thickness is altered. The microcavity is illuminated with collimated, monochromatic light of wavelength *λ* in an epi-wide-field configuration using the objective of an inverted microscope. This generates an interference pattern across the cavity depending on the local optical cavity thickness, with bright fringes appearing where local thickness of the cavity is $d\approx \frac{m\;\lambda }{2n}$ , *m* being an integer number describing the mode number and *n* the effective refractive index of the cavity mode. The interference pattern is recorded through the same objective and directed to a camera. By sweeping through a range of wavelengths, a reflectance spectrum for each pixel in the field of view is obtained, with the contrast between peaks and troughs in this spectrum depending on the *Q*-factor of the cavity, i.e. on the amount of light reflected at each side of the microcavity. Using transfer-matrix modelling of the cavity reflectance and an absolute minimum distance fitting algorithm, this information can be reliably converted into the cavity thickness at each point across the microcavity. By subtracting the mean cavity thickness, this data is subsequently converted into a displacement map of the microcavity surface.

So far, the microcavities for ERISM were generally fabricated from 0.55 mm thick glass substrates and consisted of an elastomer sandwiched between two 10-15 nm thick gold films (Fig. 1(b), left). However, this design poses several challenges: First, the fabrication process is time-consuming and requires vacuum deposition tools not routinely available to researchers working on mechanobiology. Further, to ensure adequate adhesion of the top gold film, the elastomer has to be treated with an oxygen plasma prior to deposition of the gold, a process that adds further complexity. Furthermore, the sensitive gold surface is prone to mechanical defects from handling errors or when used to study forces exerted by small animals. Additionally, the high reflectance and the optical losses introduced by absorption of the gold layers make the combination of ERISM with other optical techniques, such as fluorescence microscopy or Brillouin microscopy [31], more challenging.

To overcome these limitations, we developed a new microcavity design shown in Fig. 1(b) (right). The significant modification is the removal of the gold layers and the replacement of the elastomer material with one that shows a higher refractive index while still maintaining physiologically relevant stiffness as required for detecting deformations caused by cellular forces. In the present work, we use a commercially available phenyl-siloxane-based polymer (marketed by CHT under the name QGel 920) that undergoes moderate crosslinking in a platinum-catalyzed addition reaction between two precursor mixtures and has a refractive index of ≈ 1.50 (see ellipsometry data in Supplementary Note 2), compared to ≈ 1.41 for the previously used elastomer (marketed as Nusil 8100 by Avantor). The refractive index difference between the elastomer and the surrounding media, such as air (*n* = 1), water (*n* = 1.33), or culture medium (*n* ≈ 1.34), is sufficient to enable partial reflection of light (Fresnel reflection), as illustrated in the right cavity design of Fig. 1(b). To obtain a similar level of reflectance at the bottom interface of the elastomer layer, a thin layer of a high index metal oxide (Ta_2_O_5_) is inserted between the glass substrate and the elastomer. (Using gold at the bottom interface of the elastomer is avoided here to maximize the total transmission of the structure.) In this configuration, the contrast between peaks and troughs in the reflectance spectra is notably enhanced compared to what would be achievable using the lower-index elastomer employed in a configuration without gold mirrors for all thicknesses of Ta_2_O_5_ (Fig. 1(c)). This improved contrast is crucial to accurately and quickly detect and assign the resonance wavelengths of the structure at each point in the field of view and hence obtain an accurate deformation map. If the contrast between peaks and troughs becomes too small, noise from image acquisition or other factors can lead to erroneous or incomplete deformation maps. To obtain the maximal interference contrast as calculated for Fig. 1(c), a thickness of 80 nm Ta_2_O_5_ was chosen. Besides avoiding the need for thin films of gold, the new design makes the processing on thinner, more fragile glass substrates easier (e.g., #1.5 cover glasses with ≈ 0.17 mm thickness), as it does not require post-processing following the elastomer application *via* spin-coating. This simplifies the use of a wider range of objectives, improving light collection efficiency and resolution, especially in fluorescence imaging. However, it should be noted that the overall interference contrast is decreased to approximately 45% of what was achieved with the traditional gold mirror-based design. We found that this contrast is still sufficient for most ERISM measurements, and for experiments that are very sensitive to measurement noise, a careful choice of measurement parameters, such as acquisition time and binning can help to compensate for the decrease in contrast.

Figure 1(d) shows a representative phase contrast microscopy image of an NIH-3t3 cell attached to a high-index microcavity, while Fig. 1(e) presents the corresponding interference image obtained when the cavity is illuminated with monochromatic light. Figure 1(f) displays the change in reflectance of the microcavity as the illumination wavelength is scanned from 550 to 750 nm. By analyzing the reflectance-versus-wavelength data at each pixel in the field of view using the previously described algorithm, a map of the cell-induced substrate displacement is generated (Fig. 1 g). The map reveals an upward deformation up to 600 nm beneath the cell center and localized downward deformations of similar magnitude at the cell periphery.

### 3.2. AFM characterization

**Fig. 2.**
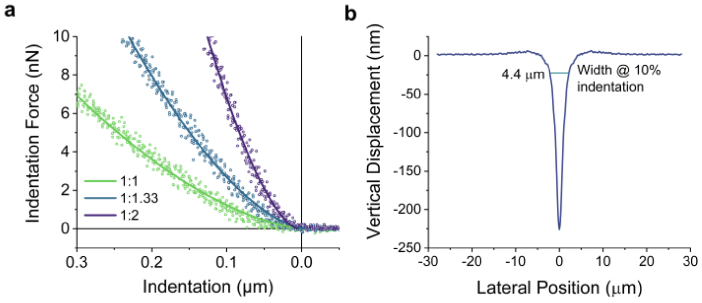
**AFM measurements. a,** Force-distance curves (open symbols) obtained by AFM micro-indentation using a 21.3 µm-diameter bead and corrected Hertz model fits (solid lines) for high-index microcavities fabricated with different elastomer mixing ratios of the elastomeric parts A and B of 1:1, 1:1.33, and 1:2 (A:B). The corresponding stiffness values derived from the fits are 5 kPa, 12 kPa, and 34 kPa, respectively. **b**, Vertical displacement profile of a high-index microcavity with a stiffness of 5 kPa, in response to indentation with a tipped AFM cantilever. The horizontal marker indicates the width of the indentation cone at 10% of the maximum indentation.

Next, we quantified the mechanical properties of the high-index microcavities using atomic force microscopy (AFM). We expect that the stiffness will depend on the mixing ratio between the two elastomeric components, A and B, used in the fabrication process (see Section 2.2) and that by increasing the ratio of component B, the crosslinker density – and consequently, the stiffness of the resulting elastomer – can be increased. To determine the stiffness of the microcavities, we applied a corrected Hertz model to fit the indentation-force/indentation-distance curves for different mixing ratios (Fig. 2(a)). For mixing ratios of 1:1, 1:1.33, and 1:2 (A:B), the corresponding stiffness values were determined to be 5 kPa, 12 kPa and 34 kPa, respectively. The sensitivity of ERISM measurements is primarily limited by the surface roughness and the noise of the optical readout, which is largely independent of the microcavity stiffness (see Supplementary Note 3). This roughness corresponds to approximately 5.9 nm, as determined by the mean standard deviation (SD) of displacements of high-index microcavities with stiffnesses of 5 kPa. For applications requiring higher sensitivity, such as detecting weak cellular contractions, the measurement noise can be reduced to 3 nm by increasing the acquisition time or by averaging over multiple measurements. In addition to resolving displacement on the nanometer scale, the new high-index microcavity is capable of measuring large indentations of up to at least 2.5 µm, provided that the indentation profile is not excessively steep (see Supplementary Note 4).

Due to the absence of surface oxidation and a gold top mirror, the new sensors are expected to show a higher spatial resolution than the gold-based sensors, i.e. have a more locally confined response to a point-like indentation force. In order to evaluate the distance over which the elastomer responds to a point-like indentation, we indented a tipped AFM probe into the sensor by 225 nm whilst detecting the deformation of the sensor with an ERISM readout (Fig. 2(b)). The width of the indentation cone at 10% of the maximum indentation depth was 4.4 µm, as indicated by the horizontal line in Fig. 2(b). By comparison, a similar measurement for a gold-based sensor showed an indentation cone with a width of 20.2 µm at a comparable indentation depth (Supplementary Note 5) [20]. This enhancement in spatial resolution may improve force mapping of small subcellular features, such as focal adhesions or invadopodia.

### 3.3. ERISM measurements on high-index microcavities

**Fig. 3.**
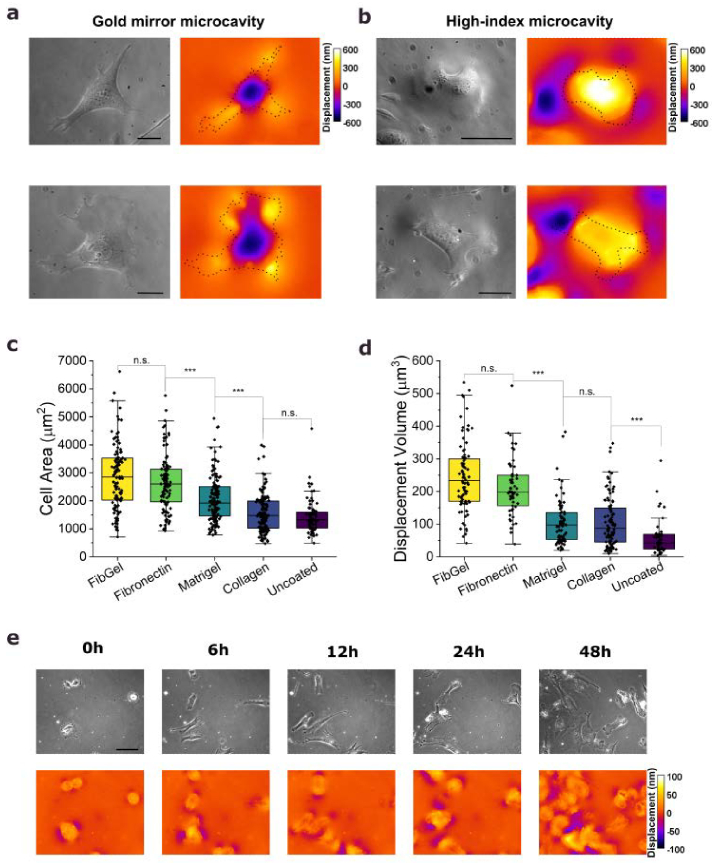
**Assessment of force patterns and cell viability. a**, **b**, Phase contrast images (left) and corresponding ERISM displacement maps (right) for representative NIH-3t3 fibroblast cells on a traditional gold mirror microcavity (**a**) and on the new high-index microcavity (**b**), each with a stiffness of ∼5 kPa. **c**, Cell area for NIH-3t3 fibroblasts cultured on high-index microcavities with an elastomer stiffness of 12 kPa and different coatings, showing median area (central line), interquartile range (IQR, box), and the minimum and maximum values within 1.5 times the IQR (whiskers). Sample sizes were as follows: collagen A (*N* = 150 cells), FibGel (*N* = 111), fibronectin (*N* = 115), Matrigel (*N* = 135), and uncoated (*N* = 72). **d**, Displaced microcavity volume per cell for cells from the same culture as in (c). Collagen A (*N* = 100), FibGel (*N* = 90), fibronectin (*N* = 58), Matrigel (*N* = 88), and uncoated (*N* = 47). **e**, Representative time-lapse of phase contrast images and ERISM displacement maps for NIH-3t3 fibroblasts on a high-index microcavity (stiffness ∼34 kPa, FibGel coating). Groups were compared using a non-parametric Mann-Whitney test for non-normally distributed data, p > 0.5 (n.s.), < 0.05 (*), < 0.01 (**), < 0.001 (***). Color scale gives elastomer displacement in nm. Scale bars, **a, b** 50µm, **e** 100 µm.

To validate the function of the new high-index microcavity design for cell measurements, we conducted ERISM experiments using both the traditional gold mirror microcavities and the new high-index microcavities, each coated with a fibronectin-gelatin mixture (FibGel, see Section 2.4). NIH-3t3 fibroblasts were used as a model cell line. Cells showed good adhesion on both structures and maps of cell-induced deformations of the cavity were readily recorded in each case. The magnitude of deformation was comparable for both cavity designs when similar stiffnesses were analyzed. For a microcavity stiffness of approximately 5 kPa, fibroblasts generated deformation depths ranging from −600 to +600 nm both on the gold mirror and the high-index microcavities (Fig. 3(a) and 3(b)).

Interestingly, the shape of the indentation profiles differs significantly between the two microcavity designs. The gold microcavity exhibits downward deformation directly beneath the cell body and upward deformation at the cell periphery, where focal adhesions anchor the cell and exert contractile forces. In contrast, the high-index microcavity displays upwards deformation in the region underneath the cell body and downwards deformation at the cell periphery outside the area of the substrate covered by the cell. This distinct deformation pattern may be attributed to the different mechanical properties of the microcavity resulting in a specific reaction to contractile forces that are oriented horizontally towards the center of the cell. The highly flexible surface of the pure elastomer in the high-index design conforms smoothly to the horizontal forces exerted by the cell and leads to an upwards deformation under the entire cell body with a counteracting downward deformation outside the focal adhesion sites. In contrast, the gold layer creates a comparatively stiff, less compliant surface that may result in a bending of the surface, comparable to the movement of a sheet of paper when it is compressed, therefore leading to a downward deformation under the cell body.

Due to the presence of phenyl moieties, the bare elastomer surface is highly hydrophobic, and a suitable bio-coating is therefore essential to maintain long-term cell viability on the new microcavities (see Supplementary Note 6). We evaluated the morphology and force generation of NIH-3t3 fibroblasts on high-index microcavities with a stiffness of 12 kPa for a series of different bio-coatings, including fibronectin, a fibronectin-gelatin (FibGel) mix, collagen A, Matrigel and a negative reference without any coating (see Section 2.4). Cell area (Fig. 3(c)) and the integrated magnitude of elastomer deformation, i.e. upwards positive displacement volume (Fig. 3(d)), show a similar trend across the different coatings, with larger displacement volumes and higher cell surface areas indicating stronger attachment to the substrate [32,33]. Cells on the FibGel-coated microcavities exhibited the strongest attachment, followed by those on fibronectin, Matrigel and collagen A coatings. Cells on uncoated microcavities showed the weakest forces and smallest cell areas, and a higher number of dead or detaching cells were observed, underscoring the importance of a suitable bio-coating for maintaining cell health. To confirm that fibroblasts indeed exhibited the largest overall force on the fibronectin-based coatings, and to exclude that the large displacement volume seen for this coating is not merely a result of the cell area being the largest, we also analyzed the maximum displacement for each cell, which is a measure of local cell force. This analysis showed the same trend as for the indented volume (Supplementary Figure S7b). Based on these results, FibGel was selected as coating for all subsequent measurements in this study. However, it is important to note that the optimal coating may differ for different cell types.

To confirm that cells remain viable on the high-index microcavities not only for the duration of a single measurement but also over extended time periods, we conducted long-term cell-force mapping for 50 hours, beginning 2 hours after seeding the cells onto the microcavity. As can be seen from the representative phase contrast images and displacement maps for different timepoints (Fig. 3(e)), as well as from the time-lapse videos ( Visualization 1 & Visualization 2, measurements every 20 minutes), cells gradually attach to the microcavity surface and apply increasingly stronger forces to the substrate, indicative of progressive formation of focal adhesions. Moreover, the time-lapse videos, including Visualization 3, which shows a 12-hour time-lapse with measurements every 10 minutes, reveal that the fibroblasts remain highly mobile and continue to divide throughout the entire measurement period. This confirms that the microcavity is a suitable substrate for long-term measurements on fibroblasts.

### 3.4. Stiffness variation of mirrorless microcavities

**Fig. 4.**
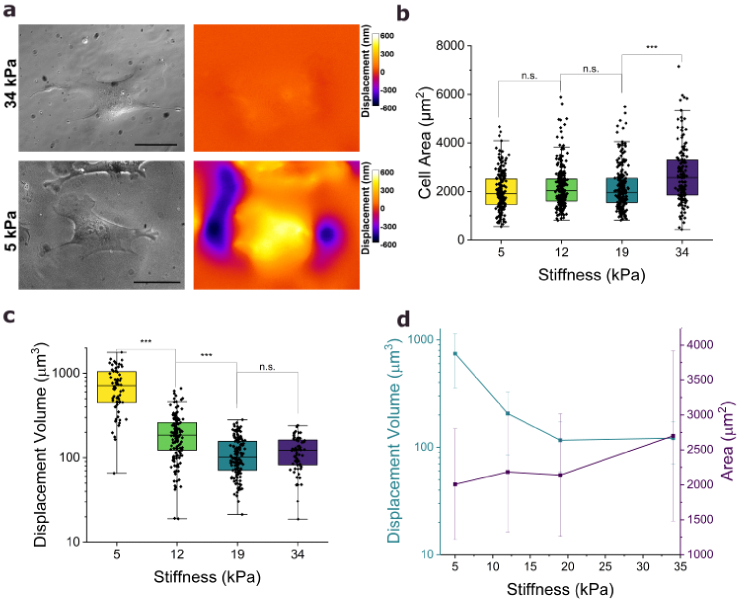
**Influence of substrate stiffness on cell behavior. a**, Transmission images (left) and displacement maps for a NIH-3t3 fibroblast on a high-index microcavity with a stiffness of either 34 kPa (top) or 5 kPa (bottom). **b**, Cell area of NIH-3t3 fibroblasts on high-index microcavities with different stiffnesses. The box plot displays the median area (central line), the interquartile range (IQR) spanning from the 25^th^ percentile to the 75^th^ percentile (box), and the minimum and maximum values within 1.5 times the IQR (whiskers). Sample sizes: *N* = 209 (5 kPa), 245 (12 kPa), 248 (19 kPa), 146 (34 kPa). **c**, Displacement volume for cells from the same culture as in panel (b). Sample sizes: *N* = 68 (5 kPa), 146 (12 kPa), 139 (19 kPa), 68 (34 kPa). **d**, Calculated mean and standard deviation of the cell areas from panel (b) and displacement volumes from panel (c) against substrate stiffness. Groups were compared using a non-parametric Mann-Whitney test for non-normally distributed data, p > 0.5 (n.s.), < 0.05 (*), < 0.01 (**), < 0.001 (***). Color scale gives elastomer displacement in nm. Scale bars, 50 µm.

Biological models exhibit diverse physiological requirements, often preferring specific substrate stiffnesses [25,34]. Since the new high-index microcavity design eliminates the need for an additional top metal layer and cells attach directly to the elastomer surface, the mechanical cell environment is largely determined by the elastomer and can be easily and precisely controlled during fabrication (see Section 3.2). The devices developed in this study exhibited a broad stiffness range, from approximately 5 kPa to 34 kPa, matching the favored physiological conditions for fibroblast cells [34,35]. As the investigated cells generally induce higher deformations on softer microcavities (Fig. 4(a)), the microcavities can be tuned for different cell model systems to maximize the visibility of applied cell forces. Furthermore, the ability to adjust stiffness without altering the optical properties or measurement parameters is particularly appealing for mechanobiological studies [1,11]. Mechanosensitive cells, including fibroblasts, adjust their physiological properties and behavior in response to the mechanical properties of their environment [3,9,36,37]. (Close observation of the map for the cell on the stiff 34 kPa substrate, for which the indentations are relatively small, shows the presence of a weak fringe-like circular step-pattern at the center of the cell. We attribute this pattern to an artifact of the fitting algorithm that becomes apparent when the cell induced deformation of the substrate is small and that likely results from parasitic thin-film interference of light within the cell body.)

As shown in Fig. 4(b) and 4(c), the cell area of NIH-3t3 fibroblasts adherent to high-index microcavities generally increases with substrate stiffness, although the variability in cell area among individual cells on the same microcavity exceeds the change in area attributed to substrate stiffness. Especially fibroblasts on the stiffest microcavity (34 kPa) show significantly increased cell areas compared to those on the softer microcavities (Fig. 4(b) and 4(c)). Although fibroblasts generally cause larger deformations to soft substrates than to stiff ones (Figs. 4(a), 4(c) and 4(d)), the extent of the displacement does not scale linearly with substrate stiffness (Fig. 4(c)). Cells on the stiffest microcavities (34 kPa) induce an even higher (although not significantly higher) displacement volume compared to those on 19 kPa substrate although the maximal measured displacement is still lower than for the softer microcavities (Supplementary Note 8). We note Gao *et al.* reported a different trend for NIH-3t3 fibroblasts, showing an increase in cell traction force for cells on substrate stiffnesses from 15 and 35 kPa and a decrease only for those on very stiff, 105 kPa substrates [38]. Together, these findings support the concept of a non-monotonic relationship between substrate stiffness and cell forces and indicate a complex adaption of fibroblasts to their mechanical environment. A reason for this behavior could be the formation of F-actin, which is triggered when cells attach to stiffer substrates or the insufficient amount of integrin on the cell membrane to exert high traction forces [36,38].

### 3.5. Integrated fluorescence and ERISM measurements

After confirming the effectiveness of the new high-index microcavity design for ERISM measurements and long-term cell viability, along with its improved tuneability of the stiffness relevant to applications in mechanobiology, we now demonstrate its enhanced performance for integrated fluorescence imaging. Simultaneous cell force and fluorescence measurements on NIH-3t3 fibroblasts were compared on a traditional gold microcavity and a high-index microcavity with a stiffness of 19 kPa. ERISM measurements were first performed on live cells, followed by fixation directly on the microcavities for fluorescence staining of vinculin, F-actin, and nuclear DNA. Epi-fluorescence imaging was carried out using the same setup as used for the ERISM measurements and the same acquisition and contrast settings were used for both the gold and high-index microcavities.

**Fig. 5.**
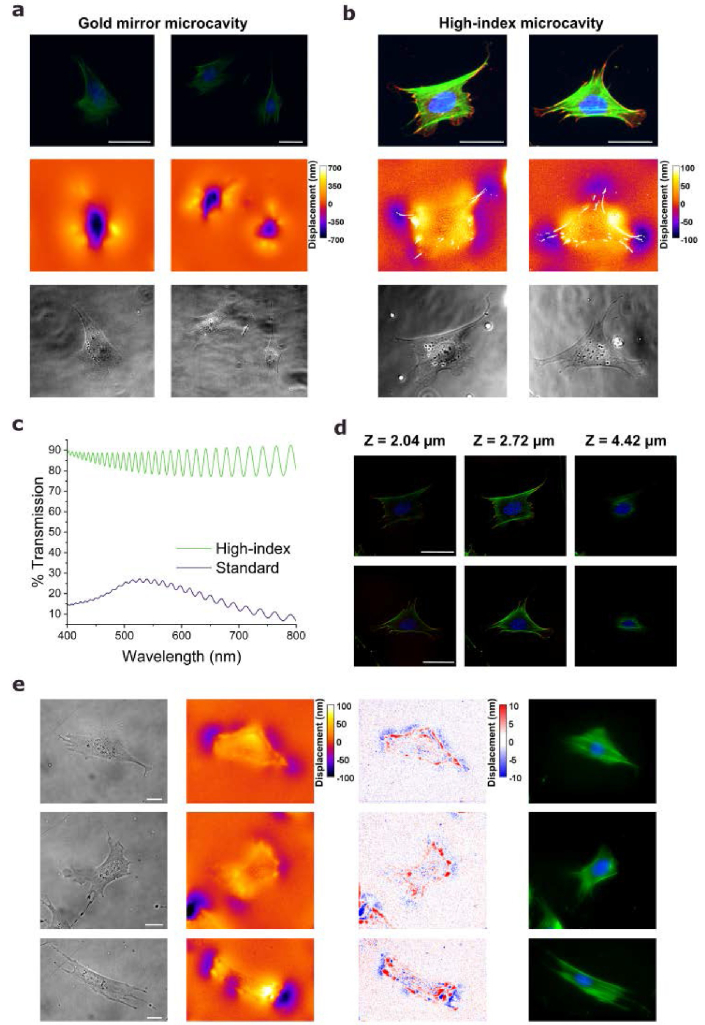
**Integrated ERISM and fluorescence measurements. a**,**b**, Epi-fluorescence images (top row), ERISM displacement maps with vinculin channel outlined in white (middle row) and transmission images (bottom row) for two representative NIH-3t3 fibroblast cells on a traditional gold microcavity (**a,** 5 kPa) and a high-index microcavity (**b**, 19 kPa). Cells were stained for actin (green), nuclear DNA (blue) and vinculin (red). ERISM measurements were conducted on live cells before fixation and staining. Acquisition settings and image processing parameters were consistent across both samples. **c**, Transmission spectra for the high-index microcavity and the standard gold mirror-based microcavity. **d**, Representative slices from confocal fluorescence z-stacks for the two cells shown in (b). The z-position of each slice is indicated for each image. **e**, Phase contrast images (left column), ERISM displacement maps in nm (second from left column), Fourier-filtered displacement maps in nm (second from right column) and epi-fluorescence images (right column) of three representative live NIH-3t3 fibroblast cells on a high-index microcavity (12 kPa). Cells were stained for actin (green) and nuclear DNA (blue). Color scale gives elastomer displacement in nm. Scale bars, 50µm.

As demonstrated in Fig. 5(a) and 5(b), the benefit of the high-index microcavities for fluorescence imaging is evident even when comparing optimized image contrast settings for each image (also c.f. Supplementary Note 9, displacement maps using same color scale are shown in Supplementary Note 10). The actin structures and nucleus are distinctly visible on the high-index microcavity, while these structures are significantly less defined on the gold mirror device, where the vinculin structure is undetectable in the same contrast settings and only barely visible with the optimized contrast settings. Although those structures have been visualized on traditional gold-based microcavities in previous studies by Kronenberg *et al* [20]., this required long acquisition times, notably slowing the measurement and increasing the light exposure of the cells, which can lead to photobleaching and cell death. The overlay of the vinculin pattern with the displacement maps (middle row of Fig. 5(b)) reveals that vinculin extends up to the points of maximum downward deformation of the cavity. The advantage of the high-index microcavities over the gold mirror-based microcavity for fluorescence imaging and other imaging techniques using epi-configuration can also been seen when comparing their transmission spectra (Fig. 4(c)). While the transmission of the gold-layered sensor is strongly wavelength dependent and only shows a mean transmission of 18% (maximum transmission, 27%), the new cavity design has a mean transmission of 85% (maximum transmission, 92%). Since the high-index microcavities were fabricated on thin glass substrates, they can also be used in confocal microscopy measurements, as these typically require the use of high numerical aperture and low working distance objectives, at least in order to deliver good quality z-stacks. This in turn provides more detailed insights into cell structures like the actin network and focal adhesion sites (Fig. 5(d), Visualization 4 and Visualization 5).

Finally, for some applications, such as long-term mapping of fluorescently labeled structures, integrated measurement of live cells might be preferable over fixed-cell imaging. The improved transparency of the high-index microcavities simplifies such measurements. By alternating between monochromatic ERISM probe light and fluorescence illumination at each cell position, real-time integrated ERISM and fluorescence imaging was achieved, as demonstrated in Fig. 5(e) and Supplementary Note 11. To visualize small, subcellular structures that interact weakly with their environment - such as podosomes - a Fourier-filtered displacement map can be calculated. For the model cell line examined in this study, the fast Fourier transform (FFT) displacement map (Fig. 5(e), third column) shows a ring-like upward deformation of approximately 10 nm at the cell periphery, accompanied by a downward deformation of similar magnitude along the boundary of this structure. These fine-scale deformations together with the overall deformation support the findings that in 2D environments, cells predominantly anchor to the substrate through focal adhesions localized at their periphery, rather than beneath the cell body [39].

## 4. Conclusion

In this study, we introduced a new microcavity design for ERISM measurements that utilizes a high-refractive-index elastomer, eliminating the need for a top metal layer. This innovation significantly simplifies and accelerates the fabrication process. The stiffness of the device can be finely tuned by adjusting the mixing ratio of the two monomeric components of the elastomer before crosslinking.

The effectiveness of this new design was validated using NIH-3t3 fibroblasts. The resulting substrate deformation patterns were found to be distinct from those observed when using gold-based microcavities, which we attribute to the transition from a more rigid metal surface of the cavity to a highly flexible elastomer surface. The absence of the top metal layer also led to a higher spatial resolution as seen by a more localized response to the point-like indentation from an AFM cantilever.

The reduced reflection and absorption of the high-index design is a further advantage, improving the compatibility of ERISM with fluorescence microcopy. High-index microcavities allow the imaging of fluorescently-labeled structures with superior resolution, and enabled not only epi-fluorescence imaging of fixed cells, but also confocal z-stack fluorescence measurements, and integrated ERISM and fluorescence measurements on live cells. Furthermore, the reduced reflection properties of the high-index microcavity open new possibilities for combining ERISM with other optical techniques, such as two-photon [40] or Brillouin [31] microscopy. Additionally, the improved transparency of the microcavity holds potential for applications in optogenetics [41,42], since light-sensitive signaling pathways could be activated through the device with reduced loss, with simultaneous mapping of cell force changes providing insights into complex biological processes. Another potential application for the high-index microcavities are animal experiments, such as investigating *Drosophila melanogaster* larva locomotion due to the improved stability and tunable stiffness of the cavity surface [43].

In summary, the high-index microcavity design enables higher-resolution mapping of cell forces and improves the integration with fluorescence microscopy while offering greater control over device properties and most importantly simplifying substrate fabrication. Furthermore, this new design presents opportunities for integrating ERISM with other optical techniques, advancing our understanding of the intricate relationship between biological processes and mechanical behavior of cells.

## Supplemental information

Supplement 1 Supplementary Information https://doi.org/10.6084/m9.figshare.30167863

Visualization 1 Time lapse video https://doi.org/10.6084/m9.figshare.30369319

Visualization 2 Time-lapse video https://doi.org/10.6084/m9.figshare.30369316

Visualization 3 12-hour time-lapse with measurements every 10 minutes https://doi.org/10.6084/m9.figshare.30369307

Visualization 4 Integrated ERISM and fluorescence measurements. https://doi.org/10.6084/m9.figshare.30369310

Visualization 5 12-hour time-lapse with measurementIntegrated ERISM and fluorescence measurements.s every 10 minutes https://doi.org/10.6084/m9.figshare.30369313

## Data Availability

Data underlying the results presented in this paper are available in Ref. [44].

## Supplemental document

See Supplement 1 for supporting content.
