# Supplementary material for: Highly transparent elastic optical microcavities for interferometric mapping of cell mechanics
Source: Biomed Opt Express. 2025 Oct 22;16(11):4617–32. doi: 10.1364/BOE.572738 (PMC12643016; doi:10.1364/BOE.572738)
Supplement: Supplement 1 [file boe-16-11-4617-s001.pdf]

Supplemental Document

Biomedical Optics EXPRESS

Highly transparent elastic optical microcavities for interferometric mapping of cell mechanics: supplement

F. Busse,[1] J. H. Booth,[1,2,3,4] N. M. Kronenberg,[1,2,3] Y. Sun,[1] A. T. Meek,[1,2,3] A. Mischok,[1] S. R. Pulver,[3,4] and M. C. Gather[1,2,3,5,*]

[1] *Humboldt Centre for Nano- and Biophotonics, Department of Chemistry and Biochemistry, University of Cologne, Cologne, Germany*
[2] *Centre of Biophotonics, SUPA, School of Physics and Astronomy, University of St Andrews, St Andrews, United Kingdom*
[3] *Centre of Biophotonics, University of St Andrews, United Kingdom*
[4] *School of Psychology and Neuroscience, University of St Andrews, United Kingdom*
[5] *Cologne Excellence Cluster on Cellular Stress Responses in Aging-Associated Disease (CECAD), University of Cologne, Cologne, Germany*
[*] *malte.gather@uni-koeln.de*

This supplement published with Optica Publishing Group on 22 October 2025 by The Authors under the terms of the Creative Commons Attribution 4.0 License in the format provided by the authors and unedited. Further distribution of this work must maintain attribution to the author(s) and the published article's title, journal citation, and DOI.

Supplement DOI: https://doi.org/10.6084/m9.figshare.30167863

Parent Article DOI: https://doi.org/10.1364/BOE.572738

Supplementary Information

Supplementary Note 1: Full schematic of the ERISM setup

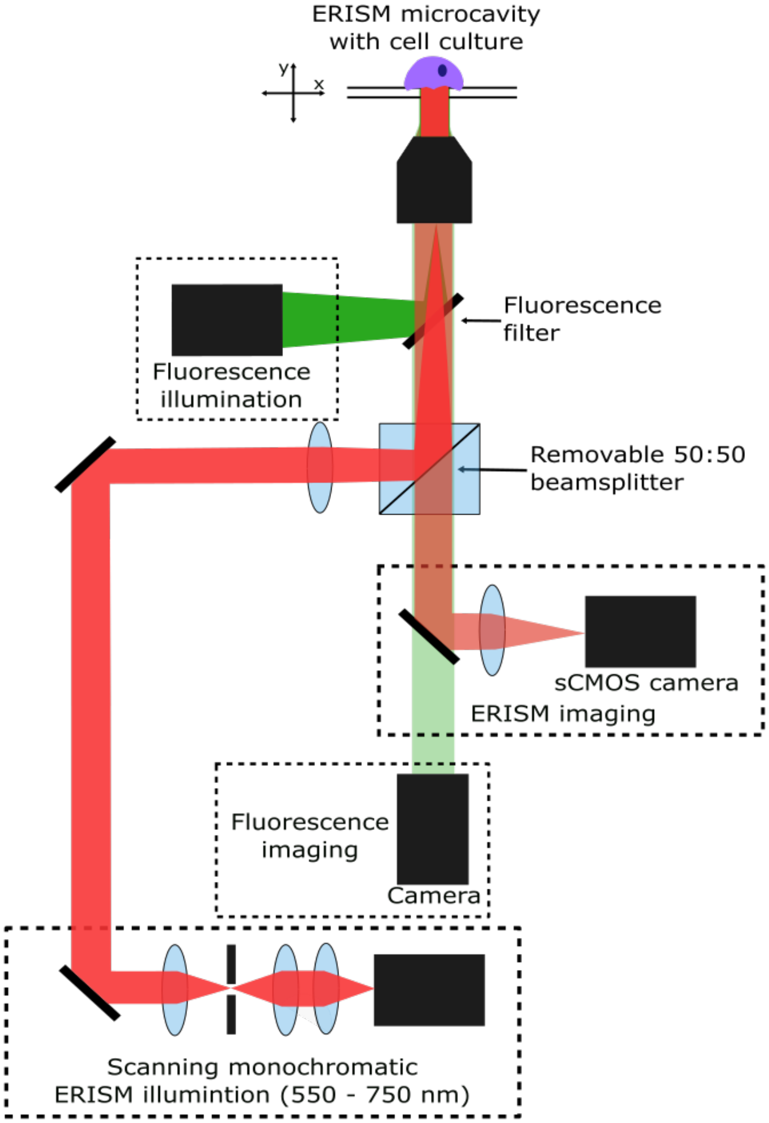

S1 | Schematic illustration of the ERISM setup integrated with a fluorescence imaging modality. The ERISM illumination (dark red) is generated by a white light source combined with a scanning monochromator and spatial filter. The light is directed upwards by a 50:50 beamsplitter and focused onto the back aperture of the objective to provide

collimated, wide-field illumination of the sample. The resulting interference signal is collected through the same objective, passed back through the 50:50 beamsplitter, and captured by an sCMOS camera. Fluorescence microscopy is integrated using a standard filter turret configuration, if required removing the 50:50 beamsplitter for maximal signal intensity.

Supplementary Note 2: Microcavity characterization

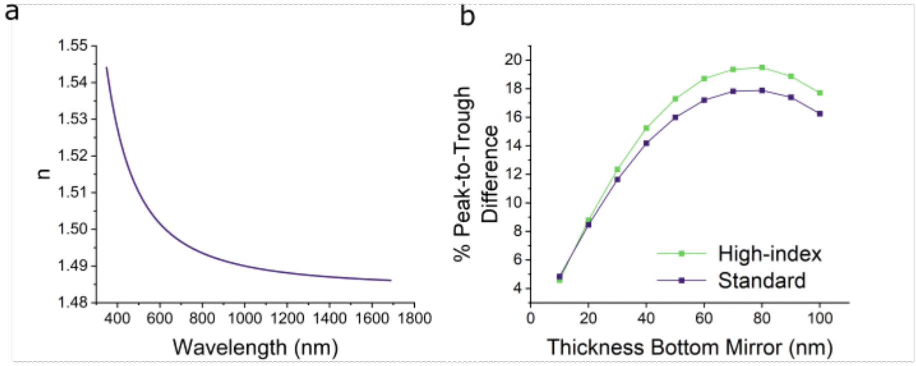

S2 | a, Refractive index *n* of the elastomer QGel920 with a stiffness of 5 kPa, as determined by ellipsometry. **b**, Mean peak-to-through contrast in the reflection spectra within the 550-750 nm wavelength range standardly used in ERISM measurements for a cavity without gold mirrors utilizing the previously used elastomer (Standard) and for the design with the high-index elastomer (High-index). Data obtained by transfer matrix modelling of reflectance spectra, assuming a 500 µm glass substrate, a Ta_2O_5 layer with variable thickness and an 8 µm thick elastomer layer.

Supplementary Note 3: Background noise from high-index microcavities

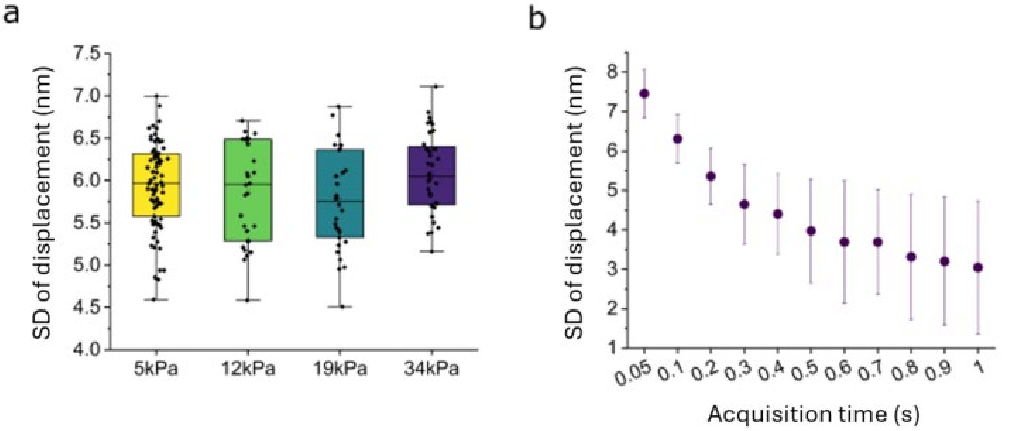

S3 | a, Standard deviation of ERISM displacements maps within areas of 5 µm x 5 µm on high-index microcavities with stiffnesses of 5 kPa, 12 kPa, 19 kPa and 34 kPa, respectively, as an estimate for the combined surface-roughness and measurement noise. The plot shows the median standard deviation (central line), interquartile range (IQR, box), and the minimum and maximum values within 1.5 times the IQR (whiskers). Sample sizes were N = 78 (5 kPa), 30 (12 kPa, 19 kPa) and 36 (34 kPa). **b**, Standard deviation of displacement maps for varying acquisitions times. Each data point in the plot shows the mean standard deviation (circles) and the 95% confidence intervals for the means (whiskers) within an area of 5 µm x 5 µm for 10 positions on a high-index microcavity with a stiffness of 5 kPa.

Supplementary Note 4: Large displacements on high-index microcavities

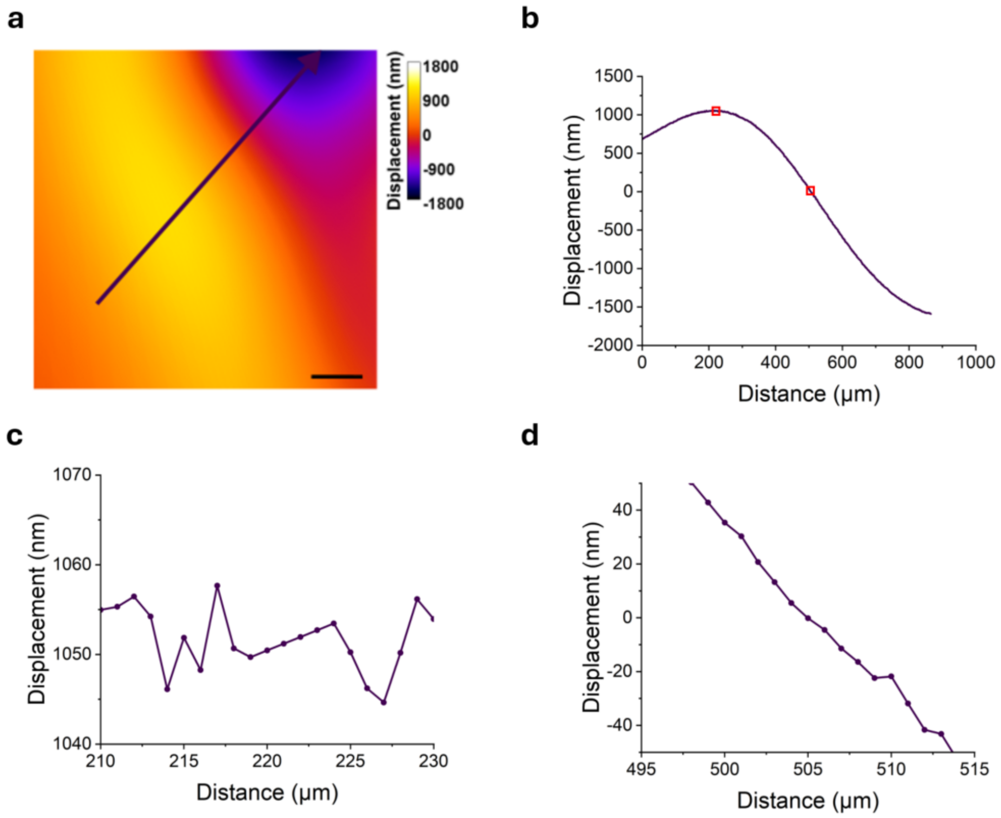

S4 | a, Displacement map for an area of high indentation on a high-index microcavity with a stiffness of 5 kPa. Scale bar, 100 µm. **b**, Displacement profile along the line indicated by the arrow in (a). A a maximal indentation of 1500 nm and a displacement range of approximately 2500 nm are resolved. Measurements were performed using a 10x objective. The plots in **c** and **d** show magnified portions of the profile for the two marked sections of the profile in (b) to highlight the accuracy of the thickness readout in different parts of the dynamic range.

Supplementary Note 5: Vertical displacement profiles

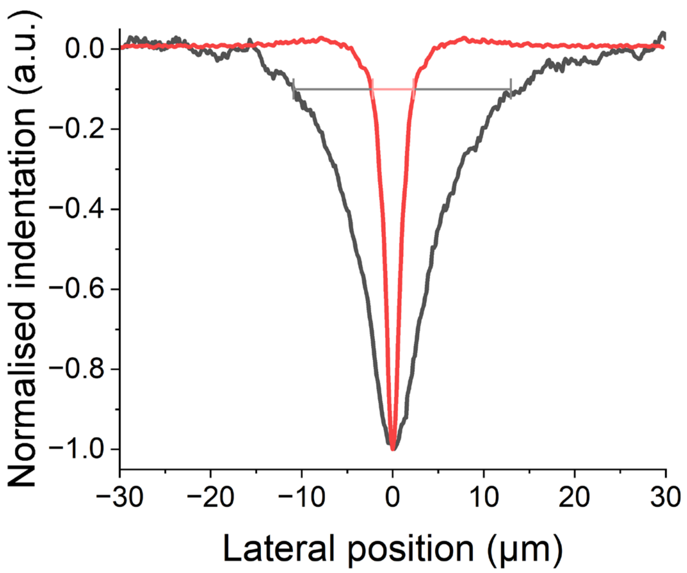

S5 | Normalized vertical displacement profile of gold mirror-based microcavity (black solid line) and a high-index microcavity (red solid line) with a stiffness of 5 kPa in response to an indentation with an AFM cantilever. Profiles were normalized to allow for an easier comparison. The absolute value of the maximum indentation was comparable in both cases, with 316 nm for the gold mirror-based cavity and 226 nm for the high-index microcavity. The profile of the gold-based cavity was taken from Kronenberg *et al.* [1], where the AFM measurement was carried out with a sphere (17 µm diameter) attached to the cantilever tip. To calculate the width of the indentation profile at 10% of the maximum indentation depth, the contact diameter of the sphere (4.6 µm at 316 nm maximal indentation) was subtracted from the value obtained from the profile (24.8 µm; gray line). AFM measurements for the high-index microcavity were carried out with a tipped AFM cantilever without a sphere attached, the width of the displacement profile at 10% of the maximum indentation is 4.4 µm.

Supplementary Note 6: Coating dependent contact angles

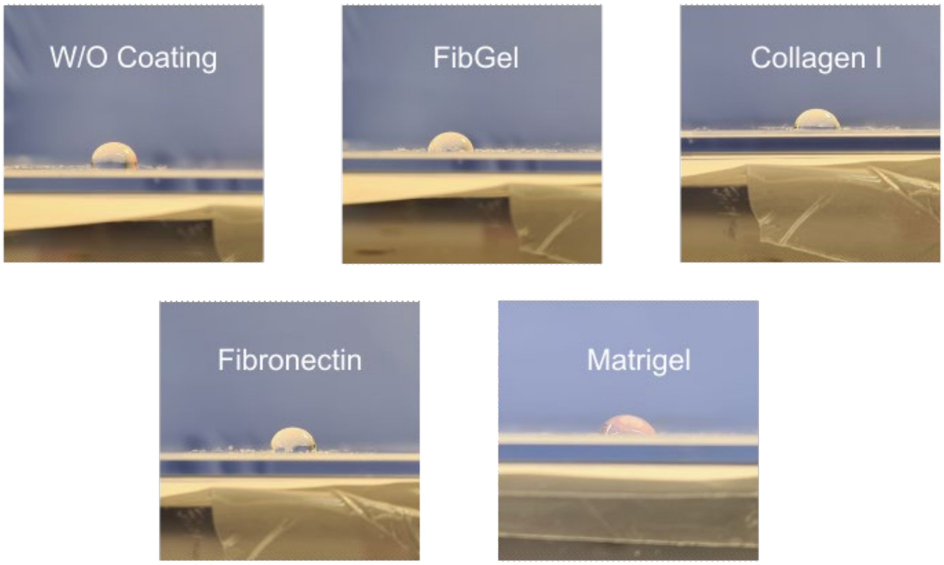

S6 | Contact angles photography of water on high-index microcavity surfaces (stiffness 5kPa). The contact angles are 100° for the uncoated microcavity, 86° for the fibronectin-gelatin (FibGel) coated microcavity, 83° for the Collagen A coated device, 91° for the fibronectin coated cavity and 66° for the Matrigel coated microcavity. Coatings were applied as described in Section 2.4 of the main manuscript. After coating, excess liquid was removed and the samples were dried at room temperature for approximately 20 minutes, except for the Matrigel coated sample, which was dried at 37°C for one hour. Contact angles were measured by placing a water droplet on the surface, with the camera aligned to ensure the droplet was centered in the focus and at the height of the microcavity surface.

Supplementary Note 7: Cell parameters on different bio-coatings

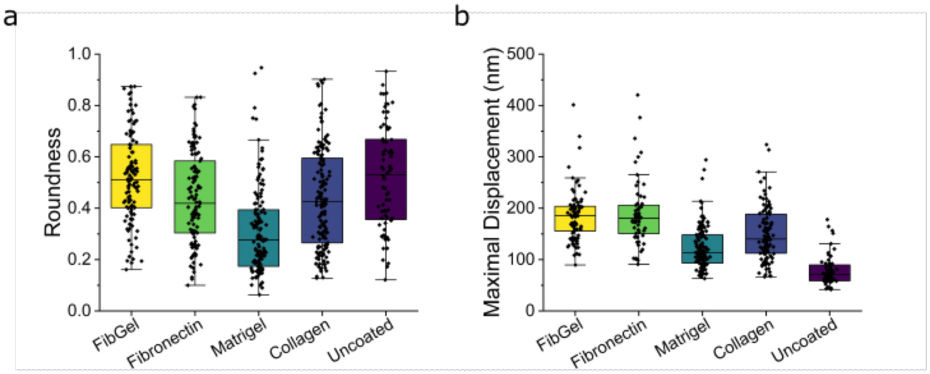

S7 | **a**, Box plots displaying the "roundness" $\left(\text{defined as } 4 \cdot \frac{\text{area}}{\pi \cdot \text{axis}^2_{\text{major}}}\right)$ of NIH-3t3 fibroblasts on high-index microcavities with a stiffness of 12 kPa and different coatings. The plot shows the median roundness (central line), interquartile range (IQR, box), and the minimum and maximum values within 1.5 times the IQR (whiskers). Sample sizes were *N*=150 (collagen A), 111 (FibGel), 115 (fibronectin), 135 (Matrigel™), and 72 (uncoated). **b**, Box plot of maximum displacement for cells from the same dish as in (a). Sample sizes were *N* = 100 (collagen A), 90 (FibGel), 58 (fibronectin), 88 (Matrigel™), and 47 (Uncoated).

Supplementary Video 1 and 2: Long-term ERISM measurements

ERISM displacement maps and phase contrast images of NIH-3t3 fibroblasts for two distinct regions on a high-index microcavity (stiffness 34 kPa). The cells in Supplementary Video 1 correspond to those in Figure **3e** of the main manuscript. The measurement was started two hours after cell seeding onto the microcavity to allow cells to settle on the surface and simultaneously capture the early attachment process. The focus was adjusted after 18 hours to accommodate complete cell settlement. Prior to the time-lapse measurement, a full ERISM wavelength scan (560 to 760 nm) was conducted at each position of the microcavity to determine the resonance order of the interference fringes. ERISM scans where then performed every 20 minutes over a wavelength range of 650 to 700 nm for 50 hours (151 scans in total). Phase contrast images were taken automatically following every scan.

Supplementary Video 3: Fast cell measurement

ERISM displacement maps and phase contrast images of NIH-3t3 fibroblasts on a high-index microcavity (stiffness 19 kPa) after six hours of incubation. A full ERISM wavelength scan (560 to 760 nm) was performed before the start of the time-lapse measurement to determine the resonance order of the interference fringes. Subsequently, ERISM scans with a wavelength range of 650 to 700 nm, followed by phase contrast imaging, were taken every 10 minutes for 12 hours.

Supplementary Video 4 and 5: z-Stack confocal microscopy

Confocal fluorescence microscopy z-stack slices (0 µm to 6.8 µm in 0.34 µm steps) of the NIH-3t3 fibroblasts on the high-index microcavity (stiffness 19 kPa), corresponding to the cells shown in Figure **5d** of the main manuscript. Cells were stained for actin (green), vinculin (red) and nuclear DNA (blue). Scale bars, 50 µm.

Supplementary Note 8: Cell properties on different microcavity stiffnesses

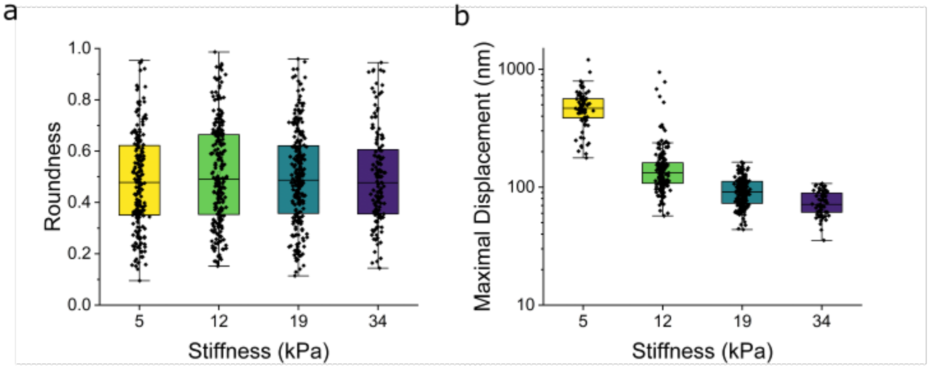

S8 | a, Box plots of the roundness of NIH-3t3 fibroblasts on high-index microcavities with different stiffnesses of 5 kPa, 12 kPa, 19 kPa and 34 kPa. The plot represents the median roundness (central line), interquartile range (IQR, box), and the minimum and maximum values within 1.5 times the IQR (whiskers). Sample sizes were N = 209 (5 kPa), 245 (12 kPa), 248 (19 kPa) and 146 (34 kPa). **b**, Box plot displaying the maximum displacement of cells from the same dish as in (a). Sample sizes were N = 68 (5 kPa), 146 (12 kPa), 139 (19 kPa) and 68 (34 kPa).

Supplementary Note 9: Fluorescence images of fixed cells

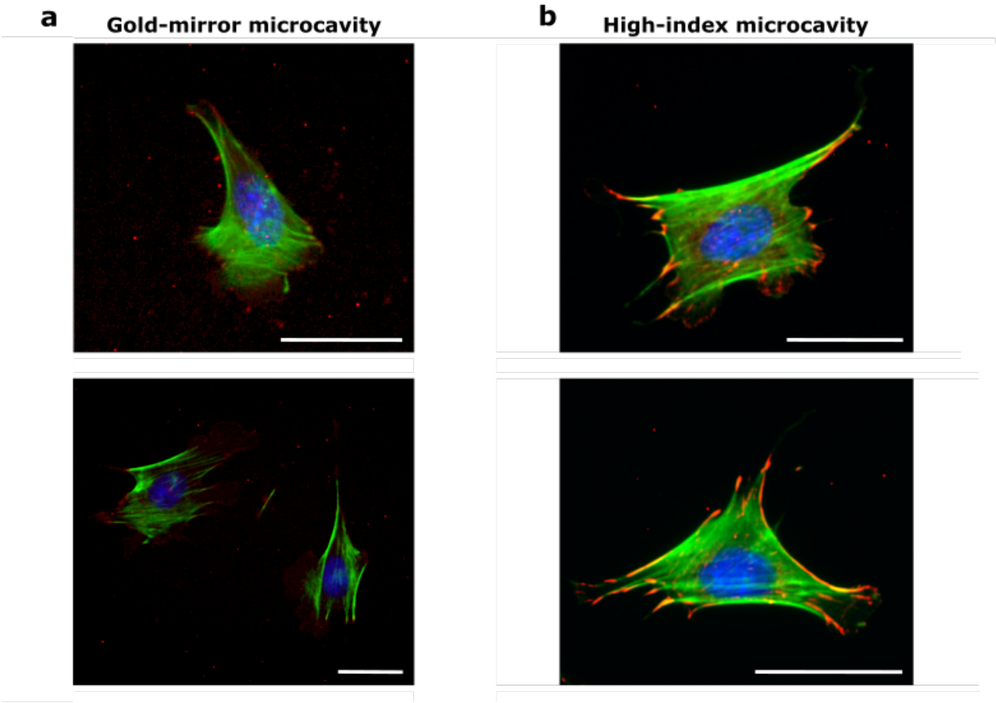

S9 | Fluorescence images of NIH-3t3 fibroblasts on a gold-mirror based microcavity (a) and a high-index microcavity with a stiffness of 19 kPa (**b**), corresponding to the fluorescence images shown in Figure **5a** and **b** of the main manuscript. Cells were imaged following fixation and staining for vinculin, actin and nuclear DNA. Acquisition settings were identical for both cavity designs, while image processing parameters were adjusted to optimize the contrast of each image. Scale bars, 50 µm.

Supplementary Note 10: ERISM displacement maps

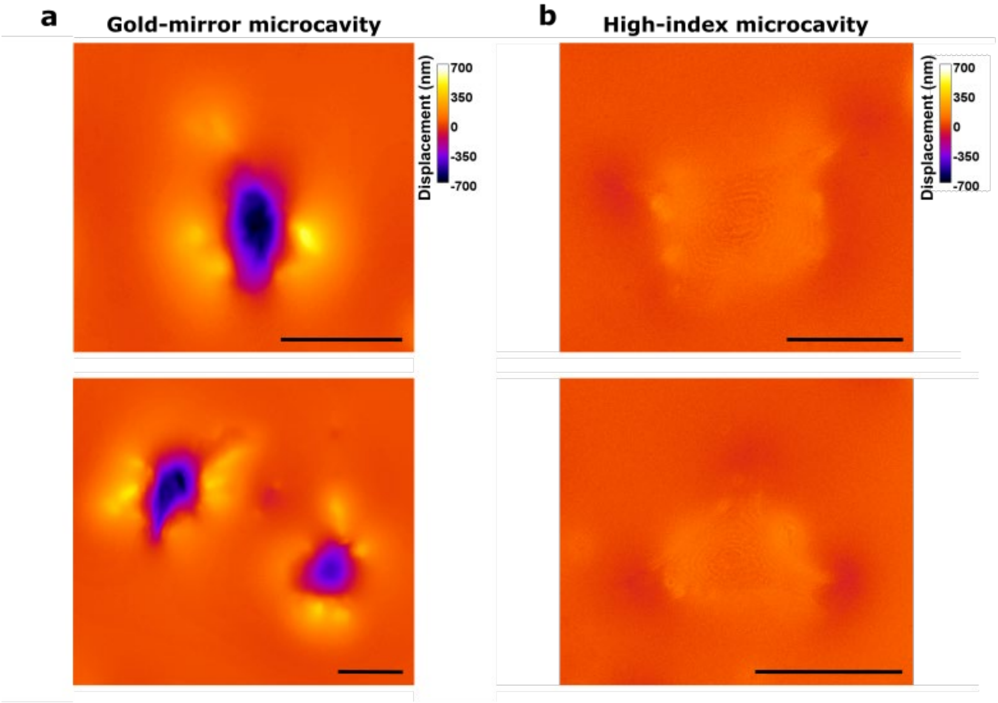

S10 | ERISM displacement maps of NIH-3t3 fibroblasts on a gold-mirror based microcavity (**a**) or a high-index microcavity with a stiffness of 19 kPa (**b**), corresponding to the ERISM displacement maps shown in Figure **5a** and **b** of the main manuscript. Scale bars, 50 µm.

Supplementary Note 11: Parallel ERISM and fluorescence imaging of live cells

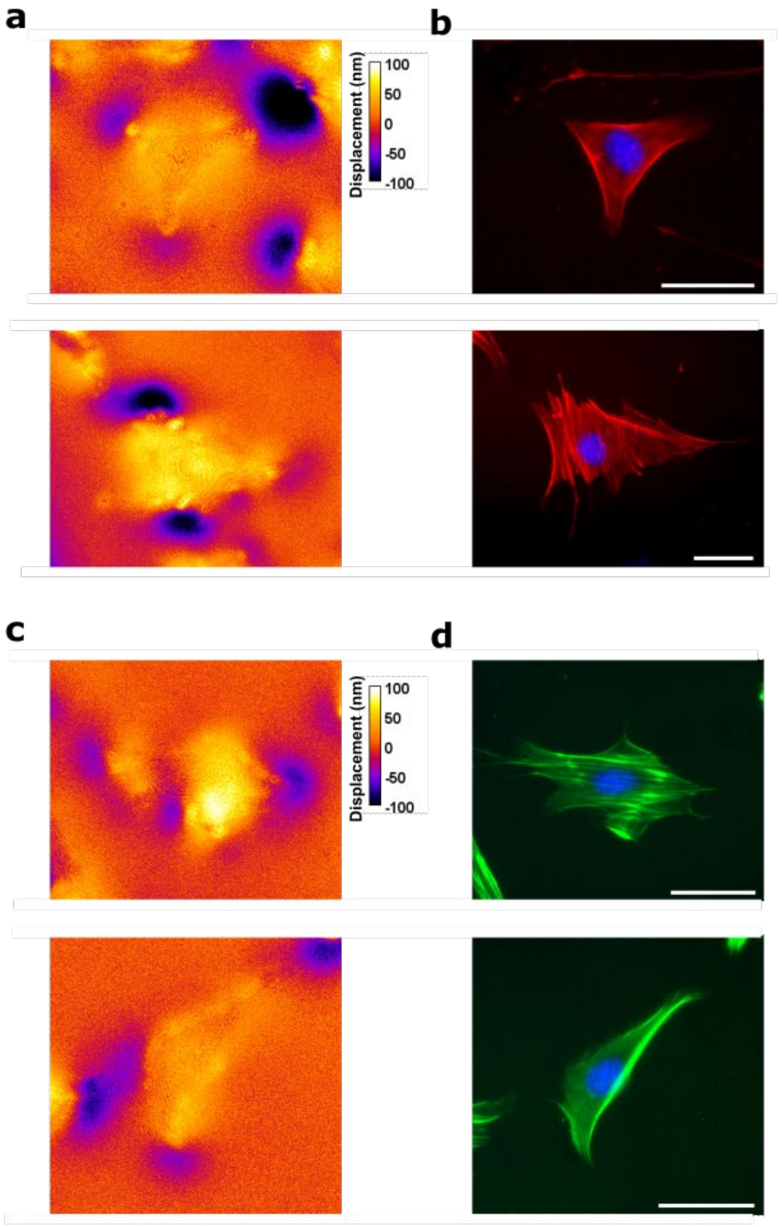

S11 | ERISM displacement maps (**a,c**) and epi-fluorescence images (**b,d**) of live NIH-3t3 fibroblasts on a high-index microcavity (stiffness, 19 kPa). Cells were stained for nuclear DNA with Hoechst and for F-actin with SiR-actin (**b**) or SPY555 (**d**). Scale bars, 50 µm.

Literature

1. N. M., Kronenberg, *et al.,* Long-term imaging of cellular forces with high precision by elastic resonator interference stress microscopy. *Nat. Cell Biol.* **19**, 864–872 (2017).
